# Population structure and phylogeography of the Gentoo Penguin (*Pygoscelis papua*) across the Scotia Arc

**DOI:** 10.1002/ece3.1929

**Published:** 2016-02-20

**Authors:** Hila Levy, Gemma V. Clucas, Alex D. Rogers, Adam D. Leaché, Kate L. Ciborowski, Michael J. Polito, Heather J. Lynch, Michael J. Dunn, Tom Hart

**Affiliations:** ^1^ Department of ZoologyUniversity of OxfordSouth Parks RoadOxfordOX1 3PSUK; ^2^ USAFAir Force Institute of Technology2950 Hobson WayWPAFBOhio45433‐7765; ^3^ Ocean and Earth SciencesUniversity of SouthamptonWaterfront CampusEuropean WaySouthamptonSO14 3ZHUK; ^4^ Department of Biology and Burke Museum of Natural History and CultureUniversity of WashingtonBox 351800SeattleWashington98195‐1800; ^5^ Department of BiologyUniversity of BristolWoodland RoadBristolBS8 1UGUK; ^6^ Department of Oceanography and Coastal SciencesLouisiana State UniversityBaton RougeLouisiana70803; ^7^ Department of Ecology and EvolutionStony Brook UniversityStony BrookNew York11794; ^8^ British Antarctic SurveyHigh CrossMadingley RoadCambridgeCB3 0ETUK

**Keywords:** Bayesian phylogeography, dispersal, microsatellites, penguins, population genetics, *Pygoscelis papua*

## Abstract

Climate change, fisheries' pressure on penguin prey, and direct human disturbance of wildlife have all been implicated in causing large shifts in the abundance and distribution of penguins in the Southern Ocean. Without mark‐recapture studies, understanding how colonies form and, by extension, how ranges shift is challenging. Genetic studies, particularly focused on newly established colonies, provide a snapshot of colonization and can reveal the extent to which shifts in abundance and occupancy result from changes in demographic rates (e.g., reproduction and survival) or migration among suitable patches of habitat. Here, we describe the population structure of a colonial seabird breeding across a large latitudinal range in the Southern Ocean. Using multilocus microsatellite genotype data from 510 Gentoo penguin (*Pygoscelis papua*) individuals from 14 colonies along the Scotia Arc and Antarctic Peninsula, together with mitochondrial DNA data, we find strong genetic differentiation between colonies north and south of the Polar Front, that coincides geographically with the taxonomic boundary separating the subspecies *P. p. papua* and *P. p. ellsworthii*. Using a discrete Bayesian phylogeographic approach, we show that southern Gentoos expanded from a possible glacial refuge in the center of their current range, colonizing regions to the north and south through rare, long‐distance dispersal. Our findings show that this dispersal is important for new colony foundation and range expansion in a seabird species that ordinarily exhibits high levels of natal philopatry, though persistent oceanographic features serve as barriers to movement.

## Introduction

Crucial to the study of evolution is characterizing population differentiation and understanding the mechanisms that disrupt gene flow. Population differentiation is the first step toward reproductive isolation in the classic model of allopatric speciation (Mayr 1942) and creates opportunities for local adaptation. Extrinsic barriers to dispersal are common in terrestrial environments (e.g., mountain ranges, rivers), but less obvious in the marine environment, where many taxa are highly mobile and physical barriers may only be manifested as changes in hydrography or fronts between water masses. Intrinsic factors, such as breeding asynchrony or natal philopatry, may therefore be expected to be more important in disrupting gene flow in the marine environment than in terrestrial systems. Seabirds provide useful models to investigate these mechanisms. They are tied to breeding locations annually, making them relatively easy to sample, yet they range widely during the nonbreeding season, allowing the comparison of extrinsic versus intrinsic factors in generating population differentiation. Characterizing population structure and dispersal is also critical to understanding population dynamics. This is particularly pertinent in regions undergoing rapid climate change, where large perturbations in population sizes can be expected. Whether these changes are driven by changes in demographic rates, such as survival and breeding success, or dispersal and range shifts, can further inform our understanding of the drivers of population differentiation and evolution.

**Figure 1:**
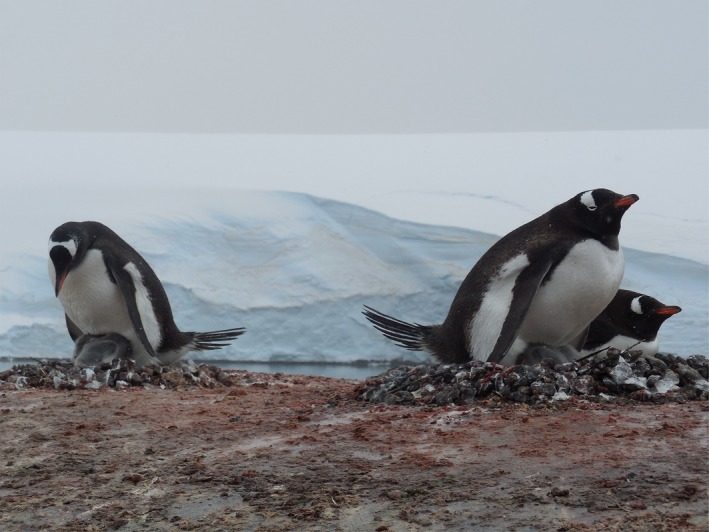
Adult Gentoo penguins (*Pygoscelis papua*) guard their chicks on the nest in the Western Antarctic Peninsula. (Photo: Hila Levy).

The Antarctic Peninsula and archipelagos lying in the Scotia Sea in the South Atlantic, better known as the Scotia Arc, are showing marked physical and ecological changes and may be experiencing some of the most rapid climate change on the planet (Vaughan et al. 2003; Fox and Vaughan 2005; Rignot et al. 2005; Ducklow et al. 2007; Mayewski et al. 2009; Cook and Vaughan 2010). Increases in sea surface temperature and changes in the extent and seasonal timing of sea ice coverage in this region (Zwally et al. 2005; Ainley et al. 2010; Lynch et al. 2012) are thought to affect predators through changes in the abundance of krill (*Euphausia superba*) (Trivelpiece et al. 2011) and breeding habitat (Fretwell and Trathan 2009; Trathan et al. 2011). The role of penguins in the Antarctic ecosystem is crucial, as they make up a large part of the avian biomass in the region, and serve as marine mesopredators. As sentinels of changes to the sensitive environments that they inhabit, penguin population dynamics are frequently used to measure the impacts of anthropogenic factors such as fishing, pollution, and global climate change (Boersma 2008). Penguins are thought to be useful indicator species because they are mobile, long‐lived predators with demographic rates that correlate strongly with environmental conditions and integrate the effects of physical and biological variability in the Antarctic environment over large temporal and spatial scales (Fraser et al. 1992; Trathan et al. 2007; Trivelpiece et al. 2011). Questions regarding gene flow and demography are important components to scientific understanding of such environmental changes. Studies have made attempts to predict the responses of penguins to such changes (Jenouvrier et al. 2009; Ainley et al. 2010), but without a mechanistic understanding of their responses and the factors that may limit their dispersal, we may be unable to make accurate predictions. Paleoecological evidence from ancient penguin colonies suggests that colonization and extinction of breeding populations have long been a part of their metapopulation dynamics (Emslie 2001; Emslie et al. 2007) and so delimiting these metapopulations using genetic techniques has been identified as a priority for future research (Chown et al. 2015). Penguins in the Scotia Arc have already shown significant changes in population sizes and ranges in concert with climate change (Woehler et al. 2001; Croxall et al. 2002; Lynch et al. 2012). This is probably not a new phenomenon, as the Antarctic ecosystem has undergone multiple fluctuations in temperature and ice extent over geological timescales. We should therefore expect penguin populations to be relatively flexible in responding to environmental fluctuations. At present, Gentoo penguin (*Pygoscelis papua*, depicted in Fig. 1) populations are increasing and moving south (Lynch et al. 2012), while Adélie and Chinstrap populations are declining in the region north of Marguerite Bay (Casanovas et al. 2015), possibly because of differential survival rather than reproductive success (Lynch et al. 2010). Understanding the genetic structure of these populations is important for interpreting whether changes in population size result from changes in local survival and recruitment or, alternatively, migration. While the southward progression of Gentoo penguins is now well documented (Fraser et al. 1992; Lynch et al. 2012), the genetic origin of new populations remains unstudied. Moreover, the observed establishment of a colony at the edge of this species’ range, at Port Lockroy on the Antarctic Peninsula circa 1985 (Trathan et al. 2008), affords us the opportunity to investigate where migrants originate, and whether migration continues to play a part in the new colony's dynamics post‐colonization. Answering this question could reveal the scale at which Gentoo penguins may exist in metapopulations, and thus the scales at which demographic models and conservation efforts should be targeted. In light of broader effects of climate change and competition for prey on faunal range expansion, understanding mechanisms of colonization is particularly important in determining the best locations for networks of protected areas to maintain population viability and genetic variation (Xu et al. 2006; Akçakaya et al. 2007). Competition with fisheries for prey such as krill and fish is concerning to marine biologists and conservationists. Competition for prey has been known to drive faunal range expansions in marine species such as grey seals (Heide‐Jorgensen et al. 1992) and terrestrial polar species (Hersteinsson and MacDonald 1992). Additionally, shifts in environmental conditions can drive bird and insect species to expand their geographic ranges (Parmesan et al. 1999; Thomas and Lennon 1999; Bennie et al. 2013). In the case of Gentoo penguins, both rapid climate change and fisheries' pressures are of concern in the Western Antarctic Peninsula, where rising temperatures and growing krill fisheries are in place. Designing an effective network of protected areas would require knowledge of patterns of colonization and movement for the species. We therefore seek to identify the population structure of Gentoo penguins across their latitudinal range and identify where the founders of a new population originated.

Gentoo penguins reside both above and below the Antarctic Polar Front, which separates the temperate climate of the Falkland Islands from the sub‐Antarctic climate of South Georgia. Further south, the climate of the South Orkney and South Shetland Islands and the western side of the Antarctic Peninsula is commonly described as maritime Antarctic (Laws 1984). There are two recognized subspecies of Gentoo penguin, and a third subspecies, recently suggested but not yet described, in the Indian Ocean (de Dinechin et al. 2012). The Northern Gentoo (*Pygoscelis papua papua*) breeds on the Falkland Islands, whilst the Southern Gentoo (*P. papua ellsworthii*) is found breeding in the Southern Ocean on South Georgia, the South Sandwich Islands, the South Orkney and South Shetland Islands and the Western Antarctic Peninsula (Stonehouse 1970; de Dinechin et al. 2012).

While they have a large breeding distribution, Gentoo penguins also exhibit high levels of natal philopatry and tend to remain within the same archipelagos year‐round (Stonehouse 1970; Tanton et al. 2004; Ratcliffe and Trathan 2011). However, determining the limits of the species’ foraging range over a lifetime is difficult because of ethical, cost, and technical challenges associated with long‐term tag or transponder use (Froget et al. 1998; Gauthier–Clerc et al. 2004; Saraux et al. 2011; Dann et al. 2014). Previous studies have indicated that Gentoo penguins have a maximum observed dispersal of 276 km during the nonbreeding season in the Falkland Islands (Clausen and Pütz 2003) and 268 km in the South Shetland Islands (Wilson et al. 1998). When foraging to provide for their chicks in the summer brood and crèche phases, they tend to stay within 50 km (Williams and Rodwell 1992; Ratcliffe and Trathan 2011) of their breeding colony. Gentoo penguins are rarely observed far out at sea (Jehl et al. 1979; Thurston 1982; White et al. 2002), although individuals have been observed as far as 2000 km from the nearest potential breeding point (Voisin 1979; Enticott 1986), indicating that long‐distance dispersal is physically possible even if its impact on genetic structuring is poorly understood. Having discrete nonbreeding habitats with large stretches of ocean between archipelagos, this species is likely to show considerable population genetic differentiation (Friesen et al. 2007a).

Mitochondrial DNA studies have revealed significant population structure in Gentoo penguins previously (de Dinechin et al. 2012; Clucas et al. 2014; Peña et al. 2014), but additional studies of population genetic structure using multiple loci are likely to play an important role in our understanding of species’ responses to environmental stressors in the polar regions by demonstrating fine‐scale levels of connectivity and dispersal barriers. For example, recent population models of Emperor penguin declines against climate change (Jenouvrier et al. 2009, 2014) have assumed populations had limited dispersal, although more recent evidence from satellites, used in high‐resolution censuses of seabird populations, suggests that colonies are more fluid than had been previously believed (Trathan et al. 2011; LaRue et al. 2015). Although the impact of these movement patterns on long‐term population projections is unknown, it is clear that even nominally site‐faithful species can have complex spatial dynamics that could influence our interpretation of mark‐recapture studies or our understanding of habitat suitability (Dugger et al. 2014). With poor resolution on the forces that have led to such wide‐ranging distribution of Gentoo penguins worldwide, we set out to use genetic markers to delineate the extent to which philopatry and oceanic barriers shape the population structure of these seabirds.

Using microsatellites taken from a range of previous studies, along with sequences of the mitochondrial hypervariable control region, we determine the population structure of Gentoo penguins across the Polar Front and around the Scotia Arc. Using fine‐scale sampling within the Falkland Island archipelago, and large‐scale sampling across the Scotia Arc region, we are able to describe the population structure in the region, while assessing the origin of a recent founder population and describe how areas were colonized postglacially. We further interpret these results in the context of recent shifts in population size and range expansion in concert with environmental change across the Scotia Arc, and implications for conservation policy.

## Materials and Methods

### Population sampling

**Figure 2:**
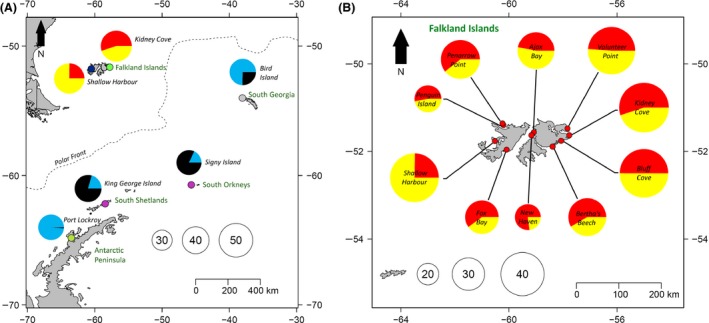
Population structure (pie charts) and locations of Gentoo penguin (*Pygoscelis papua*) breeding sites [different colors in (A), red circles in (B)] sampled in this study across the Scotia Arc. Pie chart colors denote different genetic clusters as identified by STRUCTURE, and the size of each pie slice shows the average probability of assignment of individuals to the cluster. The size of each pie chart is scaled according to the number of individuals sampled at each colony (clear circles at the bottom of each plot for scale). (A) The results of structural analysis for the whole of the Scotia Arc, which was conducted as two separate analyses (see Fig. 4B)**.** Location markers are colored according to the output of GENELAND, which found five population clusters. (B) The results from STRUCTURE for the Falkland Islands, which identified two populations (see Fig. 4B).

We collected DNA samples using a mixture of shed feathers, plucked feathers, and blood samples from locations shown in Figure 2. We collected shed feathers from 10 Falkland Island colonies (Volunteer Point, Kidney Cove, Bluff Cove, Bertha's Beach, Ajax Bay, New Haven, Fox Bay, Saunders Penguin Island, Saunders Penarrow Point, and Shallow Harbour) in May 2010 and from Port Lockroy on the Antarctic Peninsula in February 2010. At each of these sites, we took 80–125 molted penguin body and tail (retrix) feathers from nesting sites. We picked feathers from at least 2 m apart to minimize the chance of obtaining duplicate samples from an individual. All colonies sampled are known to contain at least 300 breeding pairs. We also excluded one of any pair of samples with identical mitochondrial and microsatellite genotypes. We plucked feathers from breeding birds at King George Island in the South Shetland Islands and Signy Island in the South Orkneys, and genotyped blood samples taken from adult birds in a previous study on Bird Island, South Georgia. Blood samples were drawn from the brachial vein using a 25G needle and syringe, and stored in 95% ethanol at −20°C. Feather samples were individually stored dry at ambient temperature until extraction.

### DNA extraction

Between 48 and 54 feathers from each colony were selected for extraction based on feather cleanliness and appearance. Tail feathers (retrices) were prioritized over molted body feathers, as they contain a blood supply (Williams 1995). For all feathers, approximately 5 mm of the proximal end (calamus) of the feather was finely chopped using a sterile razor blade, and deposited into a 1.5‐mL sterile microcentrifuge tube with sterile tweezers. In the case of tail feathers, the retrix was first cut down to a length of approximately 10 cm, cutting away the shaft (rachis) and leaving the superior and inferior umbilicus intact. Heavy‐duty sterile scissors were then used to cut the feather lengthwise and expose the inner pulp and blood supply. Once a dry blood vessel was found, it was scraped out of the feather casing and diced with a scalpel, and then transferred to the microcentrifuge tube with the feather tip. DNA was then extracted using the DNeasy Blood & Tissue Kit (Qiagen, Crawley, West Sussex, UK) according to the manufacturer's instructions for animal tissue, with the following user modification for the lysis step: 180 *μ*L of lysis Buffer ATL and 30 *μ*L of Proteinase K were added to the microcentrifuge tube, which was vortexed and then incubated for an extended period of 24–72 h at 56°C in a shaking incubator. Blood samples obtained from Bird Island, South Georgia, were extracted using the DNeasy Blood & Tissue Kit (Qiagen, Crawley, West Sussex, UK) according to the manufacturer's instructions for animal blood. The extracted DNA was suspended in 200 *μ*L of elution Buffer AE and stored at −20°C.

### Molecular data

Polymorphic microsatellite loci have been characterized for Galápagos penguins (*Spheniscus mendiculus*), Magellanic penguins (*Spheniscus magellanicus*) (Akst et al. 2002), Humboldt penguins (*Spheniscus humboldti*) (Schlosser et al. 2009), Little penguins (*Eudyptula minor*) (Billing et al. 2007), yellow‐eyed penguins (*Megadyptes antipodes*) (Boessenkool et al. 2008), Macaroni penguins (*Eudyptes chrysolophus*) (Ahmed et al. 2009), and closely related Adélie penguins (*Pygoscelis adeliae*), some of which have been tested for cross‐amplification in small numbers of Gentoo penguins (Roeder et al. 2001; Ahmed et al. 2009; Schlosser et al. 2009). Because of the lack of species‐specific primers in Gentoo penguins, a subset of 14 Gentoo individuals from seven colonies were screened for amplification and polymorphism using PCR primers developed for 34 microsatellite loci in other species of penguins, as well as two loci developed for petrels (Brown and Jordan 2009) (see Table 1). Amplifications were conducted in 8.5‐*μ*L volumes containing 4 *μ*L 2× Multiplex PCR Master Mix (Qiagen, Crawley, West Sussex, UK), 2 *μ*M of each primer, and 2.5 *μ*L of template DNA. Amplifications involved an initial cycle of 95°C for 15 min, 40 cycles of 94°C for 30 sec, 60°C for 90 sec, and 72°C for 45 sec, followed by a 10‐min extension at 72°C. A volume of 1 *μ*L of diluted PCR product (1:90) was then suspended in 9 *μ*L of HiDi Formamide (Applied Biosystems, Foster City, CA) with 0.3 *μ*L of GeneScan 500 LIZ size standard, and visualized on an ABI 3100 Genetic Analyzer. All but one marker amplified, although not all did so consistently. Twenty‐nine markers could be scored, with only eight loci being sufficiently variable and reliable for large‐scale analyses (after testing for linkage disequilibrium and null alleles): Ech030, Ech036, Ech050, Ech065, Ech071, Ech091 (Akst et al. 2002; Ahmed et al. 2009), Emm4 (Billing et al. 2007), and RM3 (Roeder et al. 2002). These eight markers were grouped into three multiplexes, as detailed in Table 1. Peaks were then scored for length using GeneMapper v4.0 (Applied Biosystems, Foster City, CA). In cases where peaks could not be easily scored, or there was any doubt to the identity of an allele, the sample underwent amplification and genotyping in singleplex reactions two to five additional times to minimize scoring errors.

**Table 1:** Microsatellite primer sequences used for analysis with multiplex set information, number of alleles (A), and allelic sizes and range (bp), for *n* = 510 individually genotyped Gentoo penguins (*Pygoscelis papua*)

Locus	Genbank/EMBL accession number and source	Repeat motif	Primer sequence (5′–3′) and fluoro label	Multiplex Set	A	Allele sizes observed (bp) (*n* = 510)	Obs. allele size range (bp)
Ech030	FM878361 (Ahmed et al. 2009)	(CTAT)_14_	F: [HEX]‐TGACGCCGCAGGGACTTC	1	15	264, 266, 268, 270, 272, 274, 276, 280, 284, 288, 292, 296, 300, 304, 308	264–308
			R: GCTCAGCTCTTGCTCACAGTTTCAG				
Ech036	FM878367 (Ahmed et al. 2009)	(GT)_18_	F: [HEX]‐GAGAGGGTTCAGAATGACATCACG	1	3	176, 177, 178	176–178
			R: GTCCATGGGAGCAGACCTGAG				
Ech050	FM878381 (Ahmed et al. 2009)	(AC)_12_	F: [HEX]‐TGTCCAAGTCAGCAAAGCATCC	1	4	320, 322, 324, 326	320–326
			R: CGTCTGCTGGCTGGTGAGAG				
Ech065	FM878396 (Ahmed et al. 2009)	(GT)_12_	F: [FAM]‐TGACATGTATGGGGAGGAAAGGTT	1	3	146, 152, 154	146–154
			R: ACACTGGGCCTGTGGGAAAA				
Ech071	FM878402 (Ahmed et al. 2009)	(CTCAT)_14_	F: [HEX]‐CAGCCCACCGGTCTCTTACAG	2	11	192, 197, 202, 207, 212, 217, 222, 227, 232, 233, 237	192–237
			R: TGCAATGGTCTCTTCAGGAGATG				
Ech091	FM878422 (Ahmed et al. 2009)	(GT)_9_	F: [FAM]‐TCCGCAGTTCACGAGGAGTC	2	9	406, 410, 412, 414, 416, 418, 420, 422, 431	406–431
			R: ACAAGCCCTCTGCCTGTCTTG				
Emm4	DQ837732 (Billing et al. 2007)	(CT)_12_	F: [FAM]‐GGGAGGGCCTAACAAACTAC	2	7	237, 239, 241, 243, 245, 247, 249	237–249
			R: TTAGATGCCTGGTCATTTGG				
RM3	AF289546 (Roeder et al. 2002)	(CA)_10_	F: [FAM]‐AATCAGGCTCCAAGGTCAGT	3	7	208, 211, 213, 215, 217, 219, 221	208–221
			R: ATGCAAGTGACACAAAGGCT				

For mitochondrial DNA, the hypervariable region of the mitochondrial control region (HVR1), also known as Domain I, was amplified using the primers GPPAIR3F and GPPAIR3R, as described in Clucas et al. (2014). Ten new mtDNA sequences were included plus 249 sequences from Clucas et al. (2014) with a total *n* of 259. Mitochondrial sequences were visualized using Geneious Basic v5.6.4 (Biomatters, http://www.geneious.com). Forward and reverse sequences were aligned and a consensus multiple sequence alignment was generated in Geneious and exported for further analysis.

### Genetic diversity

Micro‐checker (Van Oosterhout et al. 2004) was used to test for genotyping errors resulting from null alleles, large allele dropout, and stutter. Standard indices of genetic variability, including observed and expected heterozygosities (*H*_O_ and *H*_E_, respectively) and number of alleles, were quantified for each colony at each locus using Arlequin v3.5.1.2 (Excoffier et al. 2005). Linkage disequilibrium was tested using likelihood ratio tests with 10,000 permutations (Slatkin and Excoffier 1996). Expectations for Hardy–Weinberg equilibrium were estimated for each locus and for all loci using exact tests with 1,000,000 steps (Guo and Thompson 1992).

For microsatellites, Arlequin was used to estimate pairwise *F*_ST_'s (Weir and Cockerham 1984) and we used the SGoF+ method (Carvajal‐Rodriguez and de Uña‐Alvarez 2011) to correct for multiple hypothesis testing, using the modal method for *π*_0_ estimation and a significance level of 0.05. Arlequin was used to calculate a global *F*_ST_ using analysis of molecular variance (AMOVA). Hierarchical *F*‐statistics were then calculated to search for genetic structure and find the population grouping that maximized the among‐group variation (*F*_CT_) and minimized the variation among populations within groups (*F*_SC_) (Excoffier et al. 1992). Significance of both overall and pairwise *F*_ST_'s was computed using 1,000,000 permutations. The frequency of null alleles was estimated according to Brookfield (Brookfield 1996), and FreeNA (Chapuis and Estoup 2007) was used to determine whether null alleles were biasing estimates of population differentiation.

For the mtDNA, we calculated standard molecular diversity indices and pairwise *Φ*_ST_s in Arlequin. Molecular diversity measures and molecular distances were calculated with the Tamura and Nei substitution model and a gamma distribution (with *α *= 0.066) for rate heterogeneity among sites, as calculated in jModelTest 0.1.1 (Guindon and Gascuel 2003; Posada 2008). Pairwise *Φ*_ST_s were calculated between all colonies and significance was determined using 10,000 permutations of haplotypes between colonies.

### Isolation by distance

To test for isolation by distance among microsatellite loci, the shortest geographic distance by sea was calculated using Google Earth Pro (Google, Version 7.1.5.1557), and linearized estimates of *F*_ST_ were tested for correlation with distance using Mantel's test (Smouse et al. 1986) in R with the vegan package (Oksanen et al. 2015). Statistical significance of correlation coefficients was estimated using 10,000 permutations.

To test for isolation by distance for mitochondrial data, the correlation between these same geographic distances and pairwise *Φ*_ST_s was calculated using Mantel's test with 10,000 permutations in Arlequin.

### Population structure

We explored two approaches to derive population structure from multilocus microsatellite data. First, population structure was analyzed using STRUCTURE (Pritchard et al. 2000). We compared analyses that assumed correlated and uncorrelated allele frequencies, both with and without treating sampling locations as a priori information (Pritchard et al. 2000, 2002). For admixture model conditions, *α* was allowed to vary. The program was run with a burn‐in of 10,000 iterations, followed by 1,000,000 MCMC steps. Each value of *K* (number of populations) between 1 and 14 was run 10 times, and significance was calculated from the posterior probabilities (Pritchard et al. 2002; Evanno et al. 2005; Falush et al. 2007). The most likely value of *K* was determined using the delta *K* values from Structure Harvester (Earl and Vonholdt 2012).

Secondly, to visualize population assignment in a spatial context, we used the GENELAND package within R (Guillot et al. 2005a,b; Guillot 2008). This program incorporates GPS data for each individual (set for each breeding colony sampled) and multilocus genotype data to estimate the number of populations and the geographic boundaries between the inferred clusters. We set the number of populations from 1 to 14, varying the initial population (prior) from 1 to 14 for 1,000,000 MCMC iterations using the spatial model, testing both the correlated and uncorrelated allele frequency models.

In addition, in order to verify the presence of any confounding signal from subspecies differentiation, to test for hierarchical population structure, and to detect fine‐scale structure in a highly sampled geographic area, all analyses were repeated for the 10 Falkland Island colonies alone, and for colonies south of the Polar Front.

### Bayesian phylogeography

We estimated the ancestral locations of Gentoo penguins using a Bayesian discrete phylogeographic approach (Lemey et al. 2009) with BEAST v1.8.1 (Drummond et al. 2012). We used the mtDNA data (HVR1 region, 320 bp) for 259 penguins. To select an appropriate model of nucleotide substitution, jModelTest v2.1.6 was used (Darriba et al. 2012). We evaluated the likelihood scores for 24 substitution models, and then used the Bayesian information criterion to select the model. There were two models in the 95% confidence interval (K80 + I+G and HKY + I + G), and we used the HKY + I + G in subsequent Bayesian phylogeographic analyses. We assigned each penguin to one of five island populations: Bird Island, South Georgia (*n* = 38); Falklands (*n* = 101); King George, South Shetland Islands (*n* = 41); Port Lockroy, Antarctic Peninsula (*n* = 37); and Signy Island, South Orkney Islands (*n* = 42). We modeled island location as a discrete trait using a symmetric substitution model with the Bayesian stochastic search variable selection (BSSVS) procedure, and we reconstructed ancestral states for all ancestors. We set the clock model for the mtDNA data to a strict molecular clock. We used a coalescent tree prior with constant population size and used a normally distributed prior for the mtDNA clock rate with a mean of 0.55 and standard deviation of 0.15, based on previous calculations for the mitochondrial mutation rate in the sister species *Pygoscelis adeliae* (Millar et al. 2008). As our focus is the tree topology and the locations of ancestral populations, and not the time to the most recent common ancestor, we show a single mutation rate. However, see Clucas et al. (2014) for a greater discussion on the node ages assessed using multiple rates. The prior for locations used the approximate continuous time Markov chain rate reference prior (Ferreira and Suchard 2008). We ran the analysis for 10 million generations, sampling states every 10,000 steps. We repeated the analysis four times, checked for convergence in Tracer (Rambaut et al. 2014), and then combined the four runs using LogCombiner. We obtained a maximum clade credibility tree (MCC tree) using Tree Annotator v1.8.1.

### Population assignment

Finally, we used the microsatellite data to assign individuals to populations to determine whether there were any recent migrants within the populations that we had sampled. Assignment tests were run in Genodive v2.0b27 (Meirmans & Van Tienderen 2004). Allele frequencies that were found to be equal to zero were replaced with 0.005; 50,000 permutations of the Monte Carlo test were performed to determine the null distribution of likelihood values and the significance threshold was chosen to be 0.002 (Paetkau et al. 2004). The test statistic used was the Home Likelihood (L_h_), as we had not sampled all possible source locations for migrants.

## Results

### Genetic diversity

**Figure 3:**
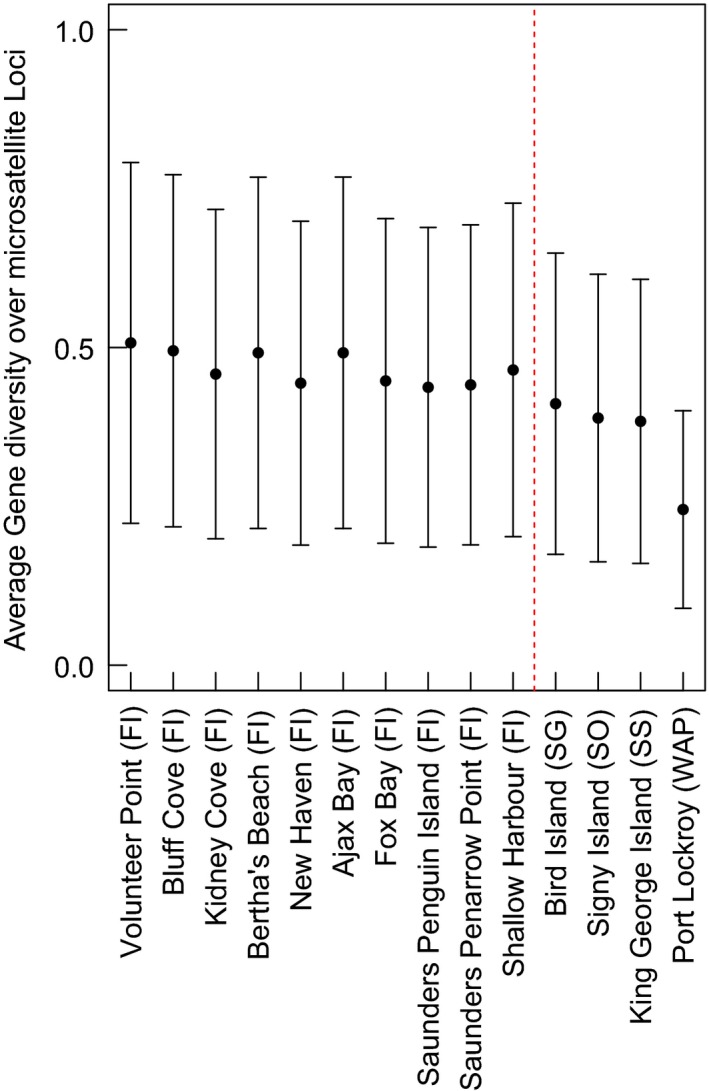
Genetic diversity of Gentoo penguins (*Pygoscelis papua*) at 14 breeding sites across the Scotia Arc. Mean observed (*H*_O_) heterozygosity is shown, along with standard deviation (SD), over eight microsatellite loci for all individuals. FI = Falkland Islands, SG = South Georgia, SO = South Orkney Islands, SS = South Shetland Islands, and WAP = Western Antarctic Peninsula. Colonies north and south of the Polar Front are shown divided by the dotted line. See also Table 2.

A total of 510 individuals were genotyped across the 14 colonies using eight microsatellite loci. These loci had between three and 15 alleles each (Table 1). Only individuals where 100% of loci could be scored were included in the analysis. None of the markers were found to be under linkage disequilibrium or to consistently deviate from Hardy–Weinberg equilibrium across colonies. Port Lockroy exhibited a slightly lower average gene diversity compared with the other colonies (*H*_O_, Table 2, Fig. 3). Locus‐by‐locus diversity measures for each sampling site are shown in Appendix S1.

**Table 2:** Genetic diversity of Gentoo penguins (*Pygoscelis papua*) at 14 breeding sites across the Scotia Arc. See Appendix S1 for diversity indices at each locus

Colony	*n*	*H*_E_	SD	*H*_O_	SD
Volunteer Point (FI)	35	0.50725	0.23398	0.39286	0.20015
Kidney Cove (FI)	46	0.45813	0.24199	0.41304	0.26472
Bluff Cove (FI)	45	0.49482	0.22322	0.48611	0.25405
Bertha's Beach (FI)	35	0.56190	0.16796	0.47347	0.17211
Ajax Bay (FI)	34	0.49166	0.23030	0.40074	0.24199
New Haven (FI)	24	0.50722	0.23709	0.45238	0.21168
Fox Bay (FI)	31	0.44738	0.24771	0.40726	0.22544
Saunders Penguin Island (FI)	25	0.49983	0.20295	0.36000	0.29029
Saunders Penarrow Point (FI)	36	0.50419	0.19574	0.42063	0.23484
Shallow Harbour (FI)	45	0.53098	0.15775	0.46667	0.23518
Bird Island (S. Georgia)	39	0.41146	0.26591	0.46795	0.29989
Signy Island (S. Orkney Is.)	37	0.38898	0.27999	0.32432	0.25559
King George Island (S. Shetland Is.)	40	0.43856	0.25379	0.46071	0.26687
Port Lockroy (Western Antarctic Peninsula)	38	0.28000	0.26133	0.28195	0.27504

Mean observed (*H*_O_) and expected (*H*_E_) heterozygosity is shown, along with standard deviation (SD), over eight microsatellite loci for all individuals (*n*). FI, Falkland Islands.

Pairwise *F*_ST_ values and associated *P*‐values are shown in Table 3. With the exception of Shallow Harbour, colonies within the Falkland Islands exhibit little genetic differentiation from one another. Although several pairs of Falkland Island colonies have significant pairwise F_ST_ values, these are all <0.05 and are several orders of magnitude lower than the differentiation seen with colonies outside of the Falklands. This significance may be attributable to natal behavior, but it should be noted that these are also the colonies with the lowest sample sizes, and which coincidentally could have been affected by demographic events such as a 2006 epidemic of avian pox on the Falklands (Munro 2006). Notably, Shallow Harbour shows consistent, moderate genetic differentiation from all colonies within the Falkland Islands (*F*_ST_ range = 0.036–0.141). All colonies within the Falkland Islands are strongly and consistently differentiated from those south of the Polar Front (*F*_ST_ range = 0.103–0.315), with strong differentiation from Signy and King George Island (*F*_ST_ range = 0.162–0.282) and from the most distant colony, Port Lockroy (*F*_ST_ range = 0.176–0.315). Of those colonies south of the Polar Front, all are strongly differentiated from one another apart from King George and Signy Island, which are not significantly differentiated from one another (*F*_ST_ = 0.008). Relative to the other Southern Gentoos, Bird Island (South Georgia) is genetically closer to, but still retains moderate to strong differentiation from, the Falkland Island colonies (*F*_ST_ range = 0.106–0.200).

**Table 3:** Pairwise *F*_ST_ values (and associated *P*‐values) among Gentoo penguin breeding sites across the Scotia Arc based on microsatellite data (below diagonal), with comparisons that are significant after correction for multiple tests (using SGoF+) shown in bold. Pairwise geographic distances (most direct distance by sea, in km) used for Mantel's test, derived from Google Earth Pro, are shown above the diagonal

	Volunteer Point (FI)	Kidney Cove (FI)	Bluff Cove (FI)	Bertha's Beach (FI)	Ajax Bay (FI)	New Haven (FI)	Fox Bay (FI)	Saunders Penguin Is. (FI)	Saunders Penarrow Point (FI)	Shallow Harbour (FI)	Bird Island (SG)	Signy Is. (SO)	King George Is. (SS)	Port Lockroy (WAP)
Volunteer Point (FI)	*	22.88	60.03	79.63	133.62	149.61	214.95	188.95	199.22	286.40	1362.08	1284.8	1207.09	1549.12
Kidney Cove (FI)	0.00864 (0.12613)	*	40.98	61.10	154.22	169.32	217.00	209.32	219.59	306.31	1349.59	1267.10	1188.79	1531.27
Bluff Cove (FI)	0.00000 (0.55450)	0.01114 (0.02762)	*	29.81	188.78	227.52	191.12	243.50	253.77	295.05	1364.64	1264.49	1171.01	1507.72
Bertha's Beach (FI)	0.00170 (0.49837)	0.01696 **(0.01564)**	0.00406 (0.24740)	*	212.99	204.02	168.91	266.56	276.83	277.87	1379.19	1263.76	1157.17	1490.56
Ajax Bay (FI)	0.00485 (0.33700)	0.02013 **(0.00693)**	0.00861 (0.09920)	0.00051 (0.55589)	*	36.15	107.33	110.52	120.71	208.92	1489.86	1402.85	1234.93	1543.97
New Haven (FI)	0.02874 **(0.00950)**	0.01841 (0.02426)	0.03672 **(0.00010)**	0.02398 (0.01634)	0.04772 **(0.00020)**	*	80.73	126.40	136.67	208.95	1507.14	1372.96	1204.72	1512.64
Fox Bay (FI)	0.01438 (0.06376)	0.00873 (0.111217)	0.00742 (0.10969)	0.00588 (0.25205)	0.00638 (0.25661)	0.02265 (0.01970)	*	197.00	211.26	194.13	1500.29	1325.39	1158.84	1463.16
Saunders Penguin Is. (FI)	0.01006 (0.21958)	0.00805 (0.19364)	0.01053 (0.10563)	0.02851 **(0.00921)**	0.02646 (0.01762)	0.04244 **(0.00287)**	0.01611 (0.08346)	*	10.27	114.81	1543.66	1490.08	1287.54	1577.65
Saunders Penarrow Point (FI)	0.01726 (0.03148)	0.01288 (0.4227)	0.01131 (0.04406)	0.01409 (0.6702)	0.01879 (0.02109)	0.02777 **(0.00842)**	0.00441 (0.31621)	0.02028 (0.04138)	*	104.96	1553.94	1498.44	1295.11	1584.72
Shallow Harbour (FI)	0.03601 **(0.00010)**	0.06855 **(0.00000)**	0.04329 **(0.00000)**	0.06189 **(0.00000)**	0.04396 **(0.00000)**	0.14086 **(0.00000)**	0.07488 **(0.00000)**	0.05963 **(0.00000)**	0.09126 **(0.00000)**	*	1640.38	1411.02	1207.17	1496.91
Bird Island (SG)	0.13304 **(0.00000)**	0.15886 **(0.00000)**	0.11542 **(0.00000)**	0.10636 **(0.00000**)	0.10258 **(0.00000**)	0.14969 **(0.00000)**	0.13988 **(0.00000)**	0.19602 **(0.00000)**	0.12443 **(0.00000)**	0.20027 **(0.00000)**	*	920.20	1476.18	1892.16
Signy Is. (SO)	0.20248 **(0.00000)**	0.22583 **(0.00000)**	0.20145 **(0.00000)**	0.19315 **(0.00000)**	0.17446 **(0.00000)**	0.22409 **(0.00000)**	0.22898 **(0.00000)**	0.28239 **(0.00000)**	0.20945 **(0.00000)**	0.26736 **(0.00000)**	0.04503 **(0.00000)**	*	686.69	1051.78
King George Is. (SS)	0.19482 **(0.00000)**	0.22237 **(0.00000)**	0.19489 **(0.00000)**	0.18340 **(0.00000)**	0.16216 **(0.00000)**	0.23258 **(0.00000)**	0.22415 **(0.00000)**	0.27731 **(0.00000)**	0.20088 **(0.00000)**	0.25087 **(0.00000)**	0.04903 **(0.00000)**	0.00793 (0.08178)	*	434.24
Port Lockroy (WAP)	0.21842 **(0.00000)**	0.26091 **(0.00000)**	0.19490 **(0.00000)**	0.17618 **(0.00000)**	0.20139 **(0.00000)**	0.27919 **(0.00000)**	0.23031 **(0.00000)**	0.31456 **(0.00000)**	0.22934 **(0.00000)**	0.25102 **(0.00000)**	0.15845 **(0.00000)**	0.27013 **(0.00000)**	0.26951 **(0.00000)**	*

FI, Falkland Islands; SG, South Georgia; SO, South Orkney Islands; SS, South Shetland Islands; WAP, Western Antarctic Peninsula. *Indicates colonies crossed pairwise with other colonies, which yields no distances

AMOVA indicated the presence of hierarchical population structure across the Scotia Arc (global *F*_ST_ = 0.117, *P *<* *0.001). The proportion of variation resulting from differences among groups was maximized at 16.38% (*P *<* *0.001) when populations were placed into four groups: (1) Falkland Island colonies; (2) Bird Island, South Georgia; (3) Signy Island, South Orkneys and King George Island, South Shetlands; and (4) Port Lockroy, Antarctic Peninsula. Explained among‐group variation remained high at 15.66% (*P *<* *0.001) when split into five groups, with Signy Island (South Orkneys) and King George Island (South Shetlands) split into separate groups, and at 15.47% (*P *<* *0.001) when Shallow Harbour (Falkland Islands) was isolated from the remaining Falkland Island colonies, as part of a five‐group hierarchy, as shown in Table 4.

**Table 4:** Analysis of Molecular Variance (AMOVA) of microsatellite data from Gentoo penguin populations of the Scotia Arc, when grouped by varying assignment criteria. The bold values indicate the grouping that maximizes among‐group variation

Grouping criteria	Within‐population % variation, *F*_ST_ (*P*‐value)	Among‐population % variation, *F*_SC_ (*P*‐value)	Among‐group % variation, *F*_CT_ (*P*‐value)
14 populations of the Scotia Arc			
1 group	88.28, 0.11721 (<0.001)		
2 groups by subspecies (Falkland Islands, Rest of Scotia Arc)	81.70, 0.18301 (<0.001)	4.62, 0.05355 (<0.001)	13.68, 0.13678 (0.001)
2 groups (Falkland Islands and South Georgia, Rest of Scotia Arc)	81.91, 0.18092 (<0.001)	6.51, 0.07359 (<0.001)	11.59, 0.11585 (0.005)
2 groups (Falkland Islands and Antarctic Peninsula, South Georgia and South Orkneys and South Shetlands)	80.43, 0.19571 (<0.001)	5.24, 0.06120 (<0.001)	14.33, 0.14328 (0.002)
3 groups (Falkland Islands, South Georgia, Rest of Scotia Arc)	82.77, 0.17225 (<0.001)	4.94, 0.05636 (<0.001)	12.28, 0.12282 (0.001)
3 groups (Falkland Islands, South Georgia and Antarctic Peninsula, South Shetlands and South Orkneys)	81.61, 0.18391 (<0.001)	3.24, 0.03822 (<0.001)	15.15, 0.15147 (<0.001)
4 groups (Falklands and Antarctic Peninsula excluding Shallow Harbour, Shallow Harbour, South Georgia, South Shetlands and South Orkneys)	83.51, 0.16492 (<0.001)	4.88, 0.05518 (<0.001)	11.61, 0.11615 (0.002)
**4 groups (Falkland Islands, South Georgia, South Shetlands and South Orkneys, Antarctic Peninsula)**	**81.29, 0.382187 (<0.001)**	**2.33, 0.02786 (<0.001)**	**16.38, 0.16382 (<0.001)**
5 groups (Falkland Islands, South Georgia, South Shetlands, South Orkneys, Antarctic Peninsula)	81.76, 0.18236 (<0.001)	2.57, 0.03048 (<0.001)	15.66, 0.15665 (0.001)
5 groups (Shallow Harbour, Rest of Falklands, South Georgia, South Shetlands and South Orkneys, Antarctic Peninsula)	83.26, 0.16737 (<0.001)	1.26, 0.01494 (<0.001)	15.47, 0.15474 (<0.001)
10 populations of the Falkland Islands			
1 group	97.26, 0.02739 (<0.001)		
2 groups (Shallow Harbour, Rest of Falkland Islands)	93.41, 0.06594 (<0.001)	1.33, 0.01407 (<0.001)	5.26, 0.05261 (0.100)
2 groups (East Falkland, West Falkland including Saunders Island)	97.21, 0.02785 (<0.001)	2.68, 0.02680 (<0.001)	0.11, 0.00109 (0.403)
3 groups by body of water (East‐facing Falkland, Falkland Sound, West‐facing Falkland)	97.26, 0.02739 (<0.001)	2.74, 0.02738 (<0.001)	0.00, 0.00001 (0.491)
3 groups by island (East Falkland, West Falkland, Saunders Island)	97.03, 0.02969 (<0.001)	2.36, 0.02376 (<0.001)	0.61, 0.00607 (0.246)

Within the Falkland Islands, very weak differentiation was present (global *F*_ST_ **= 0.027, *P *<* *0.001), with almost all variation explained by separating Shallow Harbour from the rest of the colonies (*F*_CT_ **= 5.26%), although this was not significant (*P *=* *0.100).

Haplotypic and nucleotide diversity measures for the mitochondrial DNA data are depicted in Table 5.

**Table 5:** mtDNA diversity measures for each island grouping

Grouping	*n*	*N*_H_	*N*_P_	*H* (SD)	*π* (SD)
Gentoo penguin (all)	259	115	58	0.9800 (0.0031)	0.02404 (0.01245)
*P. p. papua* (northern subspecies/all Falkland Islands)	101	40	25	0.9228 (0.0157)	0.00906 (0.00533)
*P. p. ellsworthii* (southern subspecies)	158	75	48	0.9776 (0.0047)	0.01353 (0.00746)
Bird Island (South Georgia)	38	19	18	0.9346 (0.0216)	0.00877 (0.00527)
Signy Island (S. Orkney Is.)	42	19	23	0.9338 (0.0203)	0.01041 (0.00607)
King George Island (S. Shetland Is.)	41	23	26	0.9598 (0.0150)	0.01699 (0.00929)
Port Lockroy (Western Antarctic Peninsula)	37	18	19	0.9099 (0.0300)	0.01166 (0.00670)

*n*, number of individuals sequenced; *N*_H_, number of haplotypes; *N*_P_, number of polymorphic sites; *H*, haplotype diversity; *π*, nucleotide diversity; SD, standard deviation.

### Isolation by distance

Mantel's test detected significant isolation by distance in both the microsatellite (*r *=* *0.841, *P* < 0.001) and mitochondrial (*r *=* *0.679, *P* < 0.001) regions, as might be expected over this geographic range.

### Population structure

**Figure 4:**
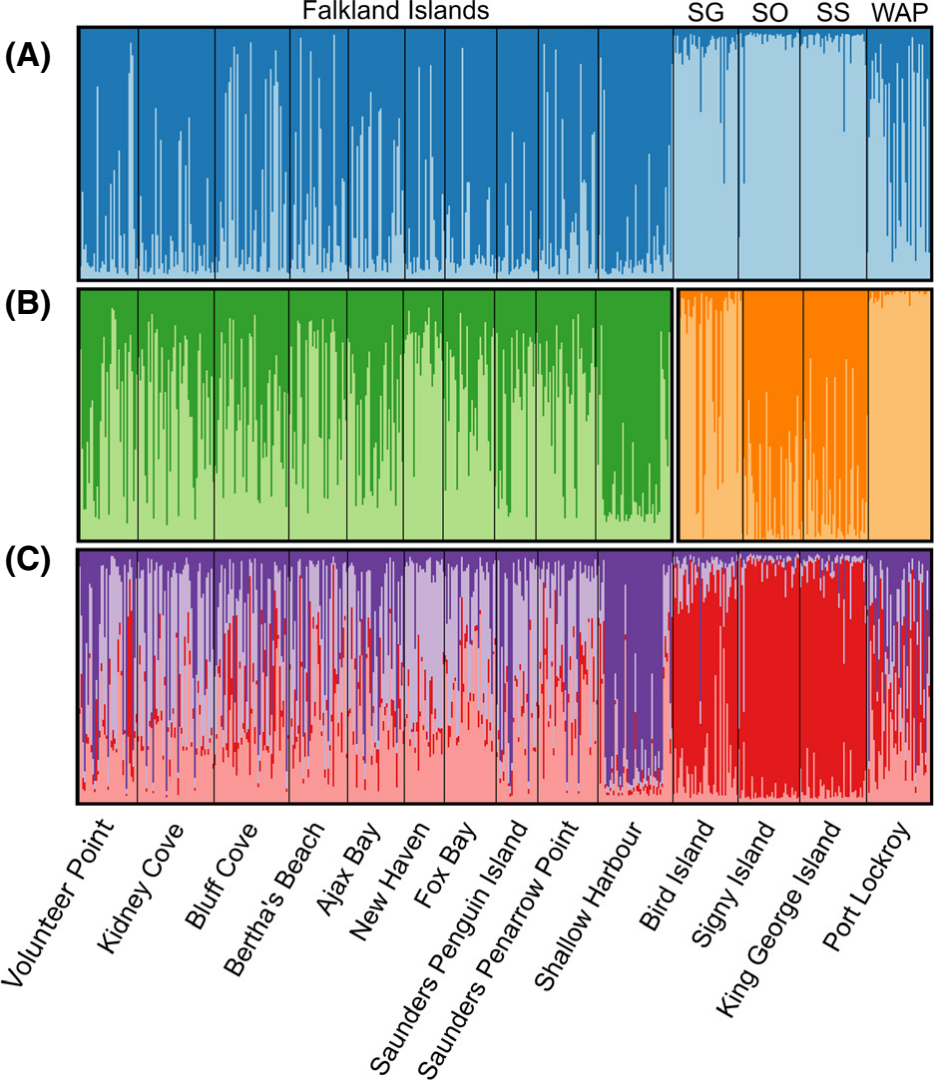
Plots of assignment probabilities from STRUCTURE showing the posterior probability of assigning each individual to each of the inferred clusters. Each individual is represented by a vertical bar, and the colors refer to the different clusters. All plots were generated from 10 runs using the admixture model with correlated allele frequencies. No location information was supplied for these runs. (A) *K* = 2 was the most likely number of clusters when all colonies were included, which clearly delineates the difference between the northern and southern subspecies of Gentoo penguin. (B) When we analyzed the northern and southern subspecies separately, *K* = 2 was most likely for each subset. (C) For illustrative purposes, we present the results from all colonies when *K* = 4, which clearly shows the differentiation of Shallow Harbour from the other Falkland Island colonies, and the difference between Northern and Southern Gentoos. SG = South Georgia, SO = South Orkney Islands, SS = South Shetland Islands, and WAP = Western Antarctic Peninsula.

The summary of results for the number of populations (*K*) inferred from STRUCTURE using Evanno's method (2005) is depicted in Table 6. The modal population when all samples were included was *K *=* *2 (Fig. 4), except under the No Admixture model for correlated allele frequencies, with no a priori location data, when *K *=* *4 was favored. The two‐population split most strongly coincided with the Northern and Southern Gentoo subspecies, with the 10 Falkland Island colonies as one population, and the four southerly colonies as another. STRUCTURE tends to detect the uppermost level of hierarchical structure in a population (Evanno et al. 2005), which may explain why *K *=* *2 was frequently reported, despite our suspicions of additional underlying structure. To delve further into the next level of hierarchical structure, we performed analyses separately on each of these two groups, which further elucidated two or three populations within the Falkland Island samples, and two or three populations within the island groups south of the Polar Front (for a total *K *=* *4–6), depending on model assumptions. Taking into account the AMOVA results, the plots for individual assignments when *K *=* *4 are displayed in Figure 4C.

**Table 6:** Summary of inferred number of populations (*K*) resulting from STRUCTURE analysis, changing model assumptions for presence of admixture, use of the LOCPRIOR setting for a priori location assignment, correlated and independent allele frequencies

Admixture or No admixture model	LOCPRIOR	Correlated or independent allele frequency model	No. of populations (*K*) inferred by Evanno method
All samples			
Admixture	Yes	Correlated	2
Admixture	Yes	Independent	2
Admixture	No	Correlated	2
Admixture	No	Independent	2
No Admixture	Yes	Correlated	2
No Admixture	Yes	Independent	2
No Admixture	No	Correlated	4
No Admixture	No	Independent	2
Falklands Only			
Admixture	Yes	Correlated	2
Admixture	Yes	Independent	2
Admixture	No	Correlated	2
Admixture	No	Independent	3
No Admixture	Yes	Correlated	2
No Admixture	Yes	Independent	2
No Admixture	No	Correlated	2
No Admixture	No	Independent	3
South of Polar Front			
Admixture	Yes	Correlated	2
Admixture	Yes	Independent	2
Admixture	No	Correlated	3
Admixture	No	Independent	3
No Admixture	Yes	Correlated	2
No Admixture	Yes	Independent	2
No Admixture	No	Correlated	2
No Admixture	No	Independent	2

GENELAND's calculation of the number of populations was strongly influenced by whether or not the correlated or uncorrelated model of allele frequencies was employed. The improved MCMC algorithm within Guillot (2008), which revisits the correlated model, seems to best explain the biological reality of our sample populations, where weak differentiation exists across most individuals, but strong differentiation is also present. The summary of GENELAND results is presented in Table 7. The resulting clustering pattern supports individual assignment to five distinct populations: (1) Shallow Harbour, Falkland Islands; (2) remaining Falkland Island colonies; (3) Bird Island, South Georgia; (4) King George Island, South Shetlands and Signy Island, South Orkneys; and (5) Port Lockroy, Antarctic Peninsula.

**Table 7:** Summary of inferred number of populations (*K*) resulting from GENELAND analysis of microsatellite data, based on both the correlated and uncorrelated models for each given prior

Prior *K*	Inferred *K* for correlated model	Inferred *K* for uncorrelated model
1	5	2
2	5	2
3	5	2
4	5	2
5	5	2
6	5	2
7	5	3
8	5	2
9	5	3
10	5	2
11	5	3
12	5	2
13	5	3
14	5	3

### Bayesian phylogeography and population assignment

**Figure 5:**
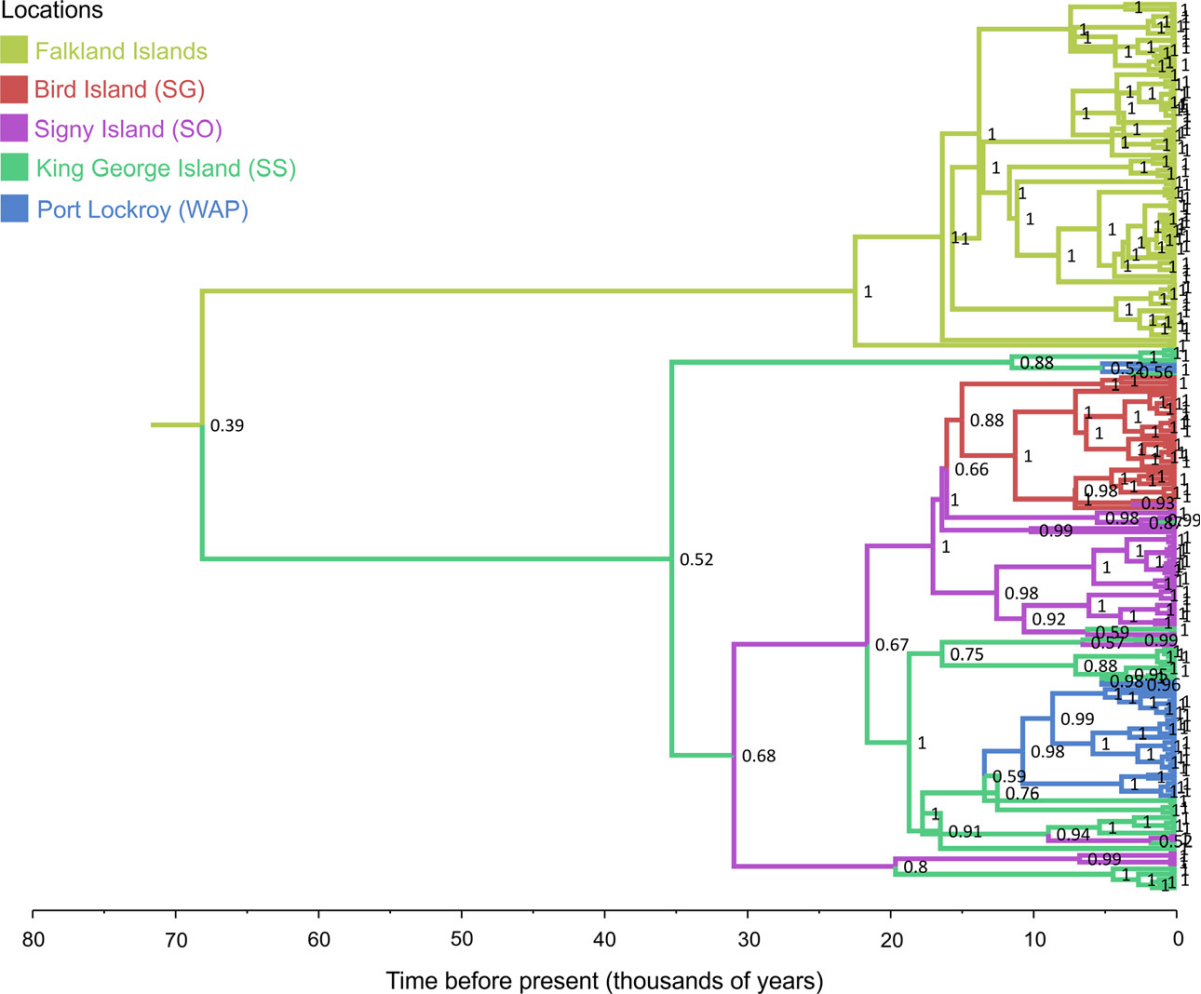
Maximum clade credibility tree derived from mtDNA showing the origin and differentiation of *Pygoscelis papua* lineages north (Falkland Islands, light green above) and south of the Polar Front (all other colors and locations). Node colors represent the most likely location of each ancestral node, whilst node labels show the level of support for each location. SG = South Georgia, SS = South Shetland Islands, WAP = Western Antarctic Peninsula, and SO = South Orkney Islands.

The maximum clade credibility tree resulting from discrete Bayesian phylogeographic analysis is depicted in Figure 5. This mitochondrial DNA tree corroborates the strong differentiation between Northern (*P. papua papua*) and Southern Gentoos (*P. papua ellsworthii*), with all Falkland individuals grouping together, as a separate clade from individuals south of the Polar Front. The Southern Gentoos seem to have radiated from a population that was in the vicinity of King George Island in the South Shetland Islands (*P* = 0.52), or possibly Signy Island in the South Orkney Islands (*P* = 0.38). From here, migrants appear to have dispersed north to South Georgia (Bird Island), with another portion of migrants moving southward from King George Island (South Shetlands) to the Antarctic Peninsula (Port Lockroy). Both the Bird Island and Port Lockroy individuals cluster to form well‐defined clades on the MCC tree, with the exception of three individuals from Port Lockroy. This suggests that populations in both South Georgia and the Antarctic Peninsula were established by a single or a small number of migration events and that ongoing gene flow has been low. As this tree is based on a single mitochondrial marker, we do not attempt to date the nodes on the tree, as more genetic loci would be necessary to draw reliable conclusions, but 95% highest posterior densities (HPDs) for node heights can be seen in Appendix S2.

Results from the population assignment tests also suggest that ongoing gene flow between those colonies south of the Polar Front has been low. None of the individuals were identified as migrants at Bird Island, King George Island, Signy Island, or Port Lockroy. However within the Falkland Islands, six individuals were identified as recent migrants between the colonies of the Falkland Islands.

## Discussion

This study has revealed that both intrinsic and extrinsic factors are important in determining gene flow in Gentoo penguins. The Polar Front, an extrinsic barrier, appears to be the most important determinant of genetic differentiation across the Scotia Arc, rather than a tendency toward natal philopatry and year‐round residency near colonies (intrinsic factors). Using both nuclear and mitochondrial markers, we have shown significant population structure in Gentoo penguins at the regional scale and explored the levels of admixture present at a finer scale within the species. The high degree of population differentiation either side of the Polar Front is consistent with similar evidence from biometric and acoustic analyses (de Dinechin et al. 2012) and mitochondrial DNA (Clucas et al. 2014), supporting the existence of two subspecies in the Scotia Arc region. There are also substantial differences in the timing of breeding between the subspecies and indeed between colonies within subspecies. Gentoo penguins have the greatest annual variation in phenology of Pygoscelid penguins. Egg laying can start as early as October in the Falkland Islands and Argentina, tends to begin in early November in the South Orkneys and South Shetlands, and extends into late November and December at Port Lockroy on the Antarctic Peninsula (Black 2015). The oceanographic barrier posed by the Polar Front, along with differences in timing of breeding (Black 2015; Bost and Jouventin 1991; Trivelpiece et al. 1987), has probably prevented noticeable admixture between Gentoo subspecies since their estimated divergence during the last glacial period (Clucas et al. 2014). The very weak genetic differentiation of populations within an archipelago, but significant population differentiation between all archipelagos, indicates that internal recruitment and survival processes probably determine population dynamics at the archipelago level, whilst a lack of long‐distance dispersal helps to maintain genetic differentiation among colonies in this philopatric seabird.

In addition, discrete Bayesian phylogeographic methods have allowed us to use mitochondrial DNA to investigate colonization patterns across island groups south of the Polar Convergence. Despite being a sex‐biased marker, the mitochondrial MCC tree shows a signal of historical radiation of Southern Gentoos from a population in the vicinity of the South Shetland or South Orkney Islands. This agrees with results from our previous work that suggested Southern Gentoos had expanded postglacially to colonize new habitat as it became available (Clucas et al. 2014). Migrants appear to have dispersed north to South Georgia, and south to the Antarctic Peninsula from this center of origin, during a limited number of migration events. The significant genetic differentiation between these regions in both the microsatellite and mitochondrial markers and the population assignment tests performed using the microsatellite data suggests that there is little ongoing gene flow to the northern and southern extremes of the Southern Gentoo's range, highlighting the role of recruitment in governing population dynamics.

It is not possible to determine whether the population in the region of the South Orkney and South Shetland Islands was a refugial population during the last glaciation or whether it was colonized soon after the Last Glacial Maximum. More molecular markers, including nuclear genes, and precise calibrations would be needed to accurately date the time to the most recent common ancestor, and hence the likely date of population splitting. Furthermore, it is also not possible to assess the likelihood of habitat being available for a glacial refuge. Bathymetry around the South Orkney and South Shetland Islands suggests that they were extensively glaciated during the last glacial period (Sugden and Clapperton 1977), but whether the coastlines were fully ice‐bound is difficult to determine, as the ancient coastlines are now submerged because of rising postglacial sea levels. The extent of historical summer sea ice in the Scotia Sea region is not well understood (Gersonde et al. 2005, Fraser et al. 2009) and so the availability of habitat for a glacial refuge is currently unclear.

Gentoo penguins are spatially segregated during summer because they are tied to their breeding sites; additional evidence suggests that they overwinter close to their summer breeding grounds and are also spatially segregated in winter (Clausen and Pütz 2003; Tanton et al. 2004; Lescroel and Bost 2005; Ghys et al. 2008; Lescroel et al. 2009). Our finding of genetic differentiation is consistent with reviews of population structure in seabirds that report that resource partitioning between populations of the same species needs to occur year‐round if it is to lead to population differentiation (Friesen et al. 2007a,b).

Some of the highest levels of population differentiation that we observed were between the Falkland Island colonies and those south of the Polar Front. This is unsurprising, given that morphological and genetic differentiation has previously been used to classify these populations into two subspecies (de Dinechin et al. 2012; Stonehouse 1970). It is notable that many genetic studies that have analyzed populations across the South Atlantic and in the Southern Ocean have detected significant genetic differentiation of populations lying either side of the Polar Front (Allcock and Strugnell 2012; Rogers 2012; Strugnell et al. 2012), but none so far in avian populations, although evidence has been found for differentiation between sub‐Antarctic and mainland New Zealand populations of yellow‐eyed penguins (Boessenkool et al. 2009). The Polar Front acts as a significant barrier to dispersal of taxa from the Antarctic to areas further north and vice versa. The reasons for this will vary by taxa, but probably reflect the marked gradient in physical conditions (mainly temperature and salinity) that extend to the seafloor, creating different biogeographic realms either side of the frontal region (Convey et al. 2012). The Polar Front has remained in its position between the Falkland Islands and South Georgia throughout the glacial history of the Antarctic (Sugden and Clapperton 1977). Habitat preferences and philopatry probably explain how this acts as a barrier to the dispersal of Gentoo penguins across the Polar Front. Ocean temperatures and prey availability differ greatly between the Falkland Islands and South Georgia (Ratcliffe and Trathan 2011), and so local adaptation could be maintaining the separation of the two subspecies. Historical factors, such as the fragmentation of populations caused by the advance and retreat of ice sheets during past glaciations and changes in the location and strength of the Antarctic Circumpolar Current, will also have played a role in creating population differentiation across many taxa (Barnes et al. 2006; Rogers et al. 2007; Strugnell et al. 2012; Chown et al. 2015). Ocean barriers, such as the Subtropical Convergence (also known as the Sub‐Antarctic Front), have obstructed gene flow in other species of penguins, including the Northern and Southern Rockhopper (*Eudyptes moseleyi* and *E. chrysocome*, respectively) (de Dinechin et al. 2009). Although currently classified as subspecies, the strong genetic differences in both nuclear and mitochondrial DNA of Northern and Southern Gentoo penguins may be indicative of incipient speciation similar to that of Rockhoppers. In a recent review, Friesen (2015) found that differences in ocean regimes, like that above and below the Polar Front, were amongst the most important factors in restricting gene for many seabirds. This disruption of gene flow has been significant enough to lead to speciation, resulting in sister‐species which occur in adjacent ocean regimes (see Friesen 2015 for a full review). Further investigation of temporal, behavioral, and spatial barriers to breeding, as well as measures of adaptation to local environments, would be needed to delve into further taxonomic elucidation of the two Gentoo groups.

While there is clear and strong differentiation between populations above and below the Polar Front, some of the most ecologically relevant differentiation exists within the Southern Ocean. Bird Island (South Georgia) emerges as a distinct population, being most closely related to the population that comprises King George Island (South Shetland Islands) and Signy Island (South Orkney Islands). Port Lockroy on the Western Antarctic Peninsula also emerges as a distinct population, again being most closely related to King George and Signy Island. It also has lower genetic diversity than any of the other populations, which is expected because of its recent establishment (*c*. 1985) (Trathan et al. 2008). Records from Charcot's expedition to the Antarctic Peninsula in 1909 suggest that there have been occasional breeders at or near Port Lockroy for at least 80 years prior to colony establishment (Charcot and Walsh 1911; Gain 1913), with established colonies observed within 35 km (Charcot and Walsh 1911). However, the area around Port Lockroy was observed to empty of breeding Gentoo penguins immediately prior to 1985 (Trathan et al. 2008).

The establishment of new colonies at the southern end of the Gentoo penguin's breeding distribution in concert with recent climate change has been interpreted as the result of local dispersal from range‐edge populations to newly suitable breeding sites just beyond (Lynch et al. 2008, 2012). Such dispersal is likely to be rare. While rapid population growth at some new Gentoo penguin colonies suggests an extended period of continuous immigration (Lynch *Unpublished data*), the maintenance of a founder population at Port Lockroy is suggestive of a single immigration event involving immigrants from outside the immediate vicinity. To have a strong founder signal, a small group of founders from the same population must have established the colony and then internal recruitment, rather than continued immigration, must have been the main driver of population growth. Populations residing along the northern portions of the Antarctic Peninsula, between Port Lockroy and King George Island, were not sampled in this study, and therefore we cannot attest to the strength of this founder effect or a potential ghost gradient that could exist in this unsampled spatial arena. However, the clustering of the majority of the Port Lockroy individuals into a clade on the mitochondrial DNA tree and the lack of detected migrants can rule out regular immigration into the Port Lockroy colony from areas outside of the Antarctic Peninsula. Future fine‐scale sampling of colonies between Port Lockroy and King George Island, as well as at additional newly established populations south of Port Lockroy, may provide additional information on the frequency and history of dispersal at smaller spatial scales.

The large population north of the Polar Front in the Falkland Islands also merits discussion. Indices of genetic diversity can assist in the elucidation of a population's recent demographic history. Overall, we see that penguins within the Falkland archipelago are interbreeding at sufficient levels to maintain similar allele frequencies and levels of overall genetic diversity across colonies. Effective population and census population size are a key component to this interbreeding. The population size of Gentoo penguins is known to have large temporal variability and interannual variation across the species range, partially accounted for by breeding abstention and deferral or low breeding success during times of low food availability (Croxall et al. 1988; Williams and Croxall 1991). This has meant that the population of Gentoo penguins in the Falkland Islands has fluctuated widely. In 1995, the estimate was 64,426 breeding pairs, followed by an increase in 2000–113,571 (79% increase). However, in November 2002, a harmful algae bloom and associated paralytic shellfish poisoning affected certain Western Falkland penguin colonies, causing mass mortality (Huin 2003). Gentoo penguin counts declined by 2005–65,857 (42% decline) following this event. Between 2005 and 2010, the population nearly doubled to 132,321 ± 2288 breeding pairs (95% increase) in the most recent comprehensive census in 2010 (Pistorius et al. 2010; Baylis et al. 2013).

Shallow Harbour, a small, west‐facing colony located on West Falkland, presents some of the most interesting signals of fine‐scale differentiation. Analytic techniques differed in their ability to find significance in the patterns of allele frequencies in this particular colony relative to the rest of the Falkland Islands. AMOVA did not show increased levels of among‐group variation when Shallow Harbour was separated from the remaining Falkland Island colonies (5 groups vs. 4 groups), although pairwise *F*_ST_ values showed significant differentiation between them. STRUCTURE visualizations show distinctions between Shallow Harbour and the other colonies. However, GENELAND analyses grouped Shallow Harbour as a separate population under the improved correlated spatial model. This could indicate that the colony underwent a recent demographic change, linked with the mass‐mortality event, which led to a deviation from Hardy–Weinberg equilibrium at four of the eight microsatellite loci assessed. Mitochondrial analysis was only performed on a very small number of individuals from this colony (primarily to discard duplicate individuals), and therefore a full assessment of mitochondrial diversity within Shallow Harbour is not possible with our data.

## Conclusions

The population genetic structure of Gentoo penguins in the Scotia Arc coincides with the oceanographic barrier presented by the Polar Front, with additional population genetic structure across the Antarctic Peninsula and on the Falkland Islands. The Polar Front appears to act as an extrinsic barrier to gene flow, even in this highly mobile seabird. We also detected a genetic signal of radiation among Southern Gentoos from King George Island, which has led to a southward founder effect at Port Lockroy. Long‐distance dispersal and colonization events appear to be rare in this species. These patterns indicate that recruitment and survival strongly influence population dynamics and that intrinsic factors such as philopatry and a tendency to remain near the breeding colony year‐round have resulted in population differentiation around the Scotia Arc. Furthermore, our findings highlight how understanding patterns of genetic diversity can help identify the demographic mechanisms influencing recent population trends in Southern Ocean predators in a time of rapid environmental change.

## Conflict of Interest

None declared.

## Data Accessibility

New mitochondrial DNA sequences will be deposited on GenBank prior to publication. Microsatellite genotyping data, along with GPS coordinates for sample sites are available from the Dryad Digital Repository: http://dx.doi.org/10.5061/dryad.84c78. The ten new mitochondrial sequences are available from Genbank with accession numbers KU527128 – KU527137. The sequences taken from our previous study can be accessed via Genbank accession numbers KJ646314 – KJ646562.

## Supporting information


**Appendix S1.** Genetic diversity of Gentoo penguins (*Pygoscelis papua*) at fourteen breeding sites across the Scotia Arc.
**Appendix S2.** Maximum clade credibility tree derived from mtDNA showing the origin and differentiation of *Pygoscelis papua* lineages north (Falkland Islands, light green above) and south of the Polar Front (all other colors and locations).
**Appendix S3.** Graph of the mean likelihood of the number of populations *L*(*K*) versus the number of populations *K*, resulting from STRUCTURE Harvester using data from all study colonies (*n* = 14), under the Admixture model with Correlated allele frequencies, using No Prior Location in STRUCTURE.
**Appendix S4.** Graph of the mean likelihood of the number of populations *L*(*K*) versus the number of populations *K*, resulting from STRUCTURE Harvester using data from all Falkland Island colonies (*n* = 10), under the Admixture model with Correlated allele frequencies, using No Prior Location in STRUCTURE.
