# Supplementary material for: Population structure and phylogeography of the Gentoo Penguin (Pygoscelis papua) across the Scotia Arc
Source: Ecol Evol. 2016 Feb 20;6(6):1834–53. doi: 10.1002/ece3.1929 (PMC4760988; doi:10.1002/ece3.1929)

**Appendix 1:** Genetic diversity of Gentoo penguins (*Pygoscelis papua*) at fourteen breeding sites across the Scotia Arc. Number of alleles (A), observed (H_O_) and expected (H_E_) heterozygosity for each locus in each population and P-values for deviations from Hardy-Weinberg Equilibrium.

| Volunteer Point (Falkland Is.) (n=35) | | | | |  | Kidney Cove (Falkland Is.) (n=46) | | | |  | Bluff Cove (Falkland Is.) (n=45) | | | |
| --- | --- | --- | --- | --- | --- | --- | --- | --- | --- | --- | --- | --- | --- | --- |
| *Locus* | *A* | *H_O_* | *H_E_* | *P* |  | *A* | *H_O_* | *H_E_* | *P* |  | *A* | *H_O_* | *H_E_* | *P* |
| Ech030 | 11 | 0.62857 | 0.84720 | 0.04558 |  | 10 | 0.69565 | 0.79646 | 0.02443 |  | 10 | 0.82222 | 0.83720 | 0.84380 |
| Ech036 | 2 | 0.45714 | 0.45714 | 1.00000 |  | 2 | 0.28261 | 0.37912 | 0.11401 |  | 2 | 0.53333 | 0.44944 | 0.31185 |
| Ech050 | 2 | 0.28571 | 0.47371 | 0.02879 |  | 3 | 0.21739 | 0.37291 | 0.00448 |  | 3 | 0.42222 | 0.44644 | 0.62981 |
| Ech065 | 2 | 0.02857 | 0.02857 | 1.00000 |  | 2 | 0.02174 | 0.02174 | 1.00000 |  | 2 | 0.02222 | 0.06517 | 0.03343 |
| Ech071 | 6 | 0.65714 | 0.68157 | 0.97782 |  | 5 | 0.69565 | 0.70258 | 0.11752 |  | 5 | 0.66667 | 0.63446 | 0.79967 |
| Ech091 | 6 | 0.31429 | 0.55238 | **0.00001** |  | 5 | 0.21739 | 0.32083 | 0.00023 |  | 5 | 0.35556 | 0.46242 | 0.15642 |
| Emm4 | 4 | 0.37143 | 0.54369 | 0.02313 |  | 4 | 0.47826 | 0.55638 | 0.21176 |  | 4 | 0.71111 | 0.62996 | 0.22142 |
| RM3 | 2 | 0.40000 | 0.47371 | 0.46982 |  | 2 | 0.69565 | 0.51505 | **0.00006** |  | 2 | 0.35556 | 0.43346 | 0.29490 |
|  | Mean H_0_ | 0.39286 |  |  |  |  | 0.41304 |  |  |  |  | 0.48611 |  |  |
|  | SD | 0.20015 |  |  |  |  | 0.26472 |  |  |  |  | 0.25405 |  |  |
| Bertha’s Beach (Falkland Is.) (n=35) | | | | |  | Ajax Bay (Falkland Is.) (n=34) | | | |  | New Haven (Falkland Is.) (n=24) | | | |
| *Locus* | *A* | *H_O_* | *H_E_* | *P* |  | *A* | *H_O_* | *H_E_* | *P* |  | *A* | *H_O_* | *H_E_* | *P* |
| Ech030 | 9 | 0.65714 | 0.83395 | 0.00485 |  | 10 | 0.73529 | 0.84065 | 0.09376 |  | 8 | 0.54167 | 0.84752 | 0.00190 |
| Ech036 | 2 | 0.42857 | 0.50559 | 0.49894 |  | 2 | 0.35294 | 0.50571 | 0.09422 |  | 2 | 0.50000 | 0.45390 | 1.00000 |
| Ech050 | 2 | 0.28571 | 0.35776 | 0.32953 |  | 2 | 0.26471 | 0.43327 | 0.03888 |  | 3 | 0.08333 | 0.08245 | 1.00000 |
| Ech065 | 1 | -- | -- | -- |  | 2 | 0.05882 | 0.05795 | 1.00000 |  | 1 | -- | -- | -- |
| Ech071 | 6 | 0.71429 | 0.74493 | 0.59896 |  | 4 | 0.76471 | 0.69227 | 0.79393 |  | 4 | 0.75000 | 0.69592 | 0.92252 |
| Ech091 | 4 | 0.25714 | 0.50890 | **0.00002** |  | 3 | 0.23529 | 0.37182 | 0.05164 |  | 3 | 0.29167 | 0.46188 | 0.00835 |
| Emm4 | 4 | 0.51429 | 0.53623 | 0.94746 |  | 4 | 0.41176 | 0.48420 | 0.52436 |  | 3 | 0.45833 | 0.52039 | 0.81258 |
| RM3 | 3 | 0.45714 | 0.44596 | 1.00000 |  | 4 | 0.38235 | 0.54741 | 0.00094 |  | 2 | 0.54167 | 0.48848 | 0.68327 |
|  | Mean H_0_ | 0.47347 |  |  |  |  | 0.40073 |  |  |  |  | 0.45238 |  |  |
|  | SD | 0.17211 |  |  |  |  | 0.24200 |  |  |  |  | 0.21168 |  |  |
| Fox Bay (Falkland Is.) (n=31) | | | | |  | Saunders Penguin Is. (Falkland Is.) (n=25) | | | |  | Saunders Penarrow Pt. (Falkland Is.) (n=36) | | | |
| *Locus* | *A* | *H_O_* | *H_E_* | *P* |  | *A* | *H_O_* | *H_E_* | *P* |  | *A* | *H_O_* | *H_E_* | *P* |
| Ech030 | 7 | 0.64516 | 0.79376 | 0.06784 |  | 9 | 0.92000 | 0.85061 | 0.11399 |  | 9 | 0.72222 | 0.79773 | 0.00655 |
| Ech036 | 2 | 0.29032 | 0.45531 | 0.05479 |  | 2 | 0.16000 | 0.21551 | 0.28696 |  | 2 | 0.50000 | 0.40689 | 0.22692 |
| Ech050 | 2 | 0.32258 | 0.27499 | 0.56760 |  | 2 | 0.08000 | 0.41143 | **0.00009** |  | 2 | 0.08333 | 0.17801 | 0.02091 |
| Ech065 | 2 | 0.03226 | 0.03226 | 1.00000 |  | 1 | -- | -- | -- |  | 1 | -- | -- | -- |
| Ech071 | 5 | 0.64516 | 0.71814 | 0.21469 |  | 5 | 0.52000 | 0.65469 | 0.18558 |  | 6 | 0.66667 | 0.66901 | 0.06225 |
| Ech091 | 3 | 0.22581 | 0.30196 | 0.29469 |  | 4 | 0.16000 | 0.40163 | **0.00039** |  | 3 | 0.19444 | 0.48083 | **0.00000** |
| Emm4 | 3 | 0.61290 | 0.56108 | 1.00000 |  | 3 | 0.40000 | 0.50694 | 0.30404 |  | 3 | 0.33333 | 0.49257 | 0.02637 |
| RM3 | 3 | 0.48387 | 0.44157 | 0.78190 |  | 2 | 0.28000 | 0.45796 | 0.07422 |  | 3 | 0.44444 | 0.50430 | 0.70292 |
|  | Mean H_0_ | 0.40726 |  |  |  |  | 0.36000 |  |  |  |  | 0.42063 |  |  |
|  | SD | 0.22543 |  |  |  |  | 0.29028 |  |  |  |  | 0.23485 |  |  |
|  |  |  |  |  |  |  |  |  |  |  |  |  |  |  |
| Shallow Harbour (Falkland Is.) (n=45) | | | | |  | Bird Island (S. Georgia) (n=39) | | | |  | Signy Is. (S. Orkney Is.) (n=37) | | | |
| *Locus* | *A* | *H_O_* | *H_E_* | *P* |  | *A* | *H_O_* | *H_E_* | *P* |  | *A* | *H_O_* | *H_E_* | *P* |
| Ech030 | 9 | 0.73333 | 0.79900 | 0.25681 |  | 9 | 0.84315 | 0.88178 | 0.07640 |  | 12 | 0.83784 | 0.89448 | 0.09943 |
| Ech036 | 2 | 0.64444 | 0.45668 | 0.00704 |  | 2 | 0.28205 | 0.24542 | 1.00000 |  | 3 | 0.02703 | 0.07960 | 0.01371 |
| Ech050 | 3 | 0.24444 | 0.57353 | **0.00000** |  | 2 | 0.17949 | 0.16550 | 1.00000 |  | 3 | 0.32432 | 0.27953 | 0.63454 |
| Ech065 | 1 | -- | -- | -- |  | 2 | 0.02564 | 0.02564 | 1.00000 |  | 2 | 0.05405 | 0.10367 | 0.08100 |
| Ech071 | 6 | 0.75556 | 0.67516 | 0.70588 |  | 6 | 0.46154 | 0.42857 | 0.72775 |  | 5 | 0.27027 | 0.31100 | 0.26213 |
| Ech091 | 5 | 0.35556 | 0.39201 | 0.17693 |  | 5 | 0.46154 | 0.47686 | 0.06045 |  | 5 | 0.32432 | 0.49056 | **0.00062** |
| Emm4 | 3 | 0.22222 | 0.38926 | 0.00150 |  | 3 | 0.84615 | 0.56743 | **0.00021** |  | 5 | 0.48649 | 0.66679 | 0.02093 |
| RM3 | 3 | 0.31111 | 0.43121 | 0.03987 |  | 3 | 0.64103 | 0.50050 | 0.06190 |  | 4 | 0.27027 | 0.28619 | 0.66324 |
|  | Mean H_0_ | 0.46667 |  |  |  |  | 0.46757 |  |  |  |  | 0.32432 |  |  |
|  | SD | 0.23518 |  |  |  |  | 0.29935 |  |  |  |  | 0.25559 |  |  |
| King George Is. (S. Shetland Is.) (n=40) | | | | |  | Port Lockroy (Western Antarctic Peninsula) (n=38) | | | |  |  | | | |
| *Locus* | *A* | *H_O_* | *H_E_* | *P* |  | *A* | *H_O_* | *H_E_* | *P* |  |  |  |  |  |
| Ech030 | 9 | 0.82500 | 0.84557 | 0.84932 |  | 9 | 0.79487 | 0.76190 | 0.00543 |  |  |  |  |  |
| Ech036 | 2 | 0.07500 | 0.07310 | 1.00000 |  | 2 | 0.02564 | 0.02564 | 1.00000 |  |  |  |  |  |
| Ech050 | 2 | 0.27500 | 0.33892 | 0.33383 |  | 2 | 0.15385 | 0.22644 | 0.09473 |  |  |  |  |  |
| Ech065 | 1 | -- | -- | -- |  | 1 | -- | -- | -- |  |  |  |  |  |
| Ech071 | 6 | 0.52500 | 0.47278 | 0.86475 |  | 4 | 0.15385 | 0.14685 | 1.00000 |  |  |  |  |  |
| Ech091 | 5 | 0.65000 | 0.55570 | 0.61187 |  | 3 | 0.51282 | 0.47519 | 0.82431 |  |  |  |  |  |
| Emm4 | 4 | 0.62500 | 0.56234 | 0.56857 |  | 4 | 0.28205 | 0.25408 | 1.00000 |  |  |  |  |  |
| RM3 | 2 | 0.25000 | 0.22152 | 1.00000 |  | 2 | 0.05128 | 0.05062 | 1.00000 |  |  |  |  |  |
|  | Mean H_0_ | 0.46071 |  |  |  |  | 0.28205 |  |  |  |  |  |  |  |
|  | SD | 0.26687 |  |  |  |  | 0.27932 |  |  |  |  |  |  |  |

**Appendix 2:** Maximum clade credibility tree derived from mtDNA showing the origin and differentiation of *Pygoscelis papua* lineages north (Falkland Islands, light green above) and south of the Polar Front (all other colours and locations). Node colours represent the most likely location of each ancestral node, whilst node labels show the 95% highest posterior densities (HPDs) for node heights (time).

**
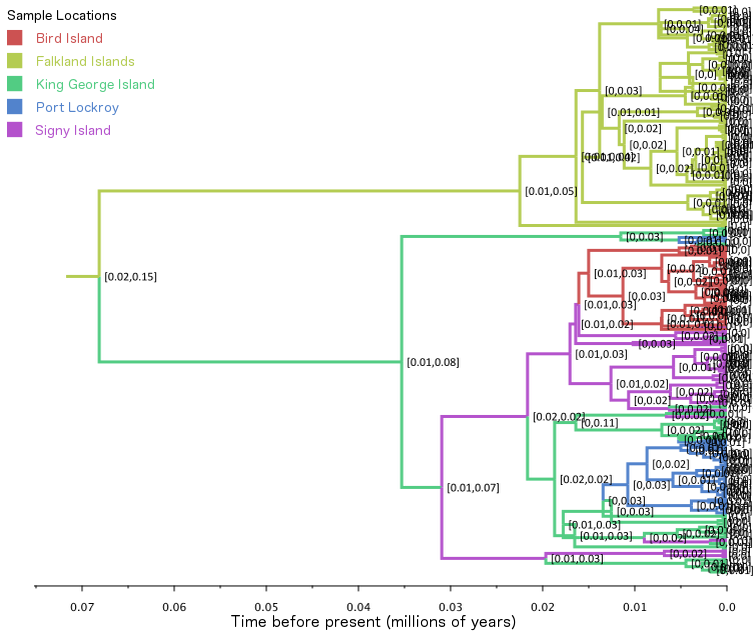
**

**Appendix 3:** Graph of the mean likelihood of the number of populations L(K) versus the number of populations K, resulting from STRUCTURE Harvester using data from all study colonies (n = 14), under the Admixture model with Correlated allele frequencies, using No Prior Location in STRUCTURE.


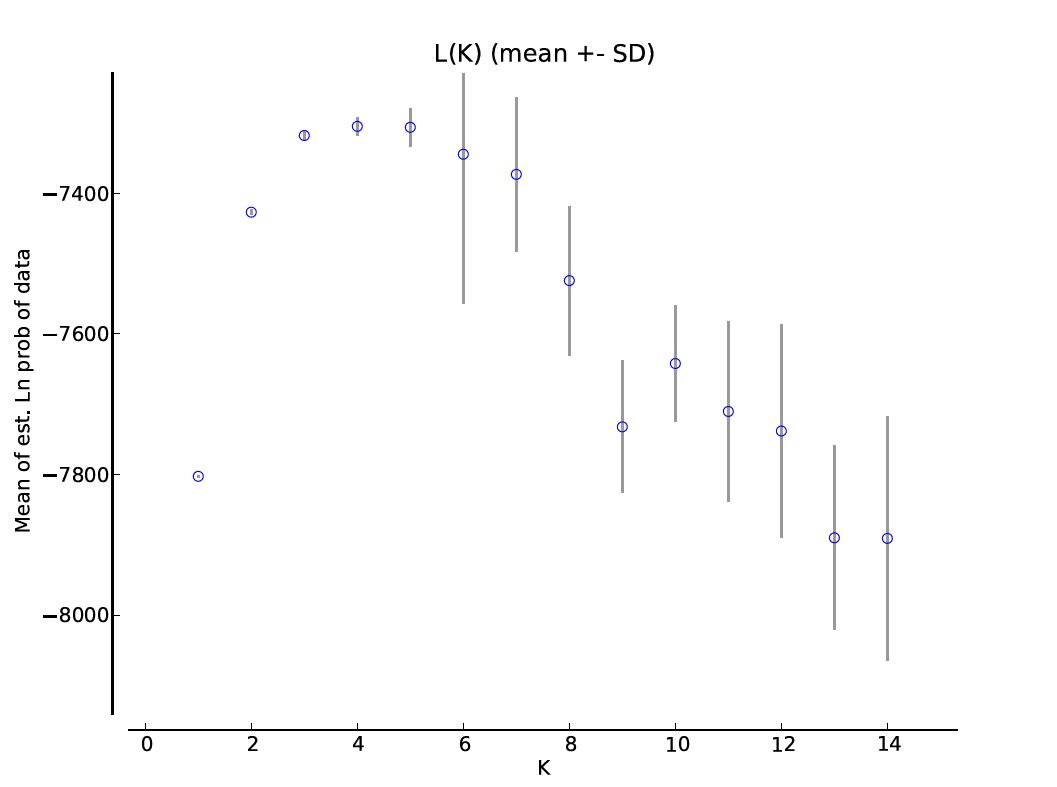


**Appendix 4:** Graph of the mean likelihood of the number of populations L(K) versus the number of populations K, resulting from STRUCTURE Harvester using data from all Falkland Island colonies (n = 10), under the Admixture model with Correlated allele frequencies, using No Prior Location in STRUCTURE.


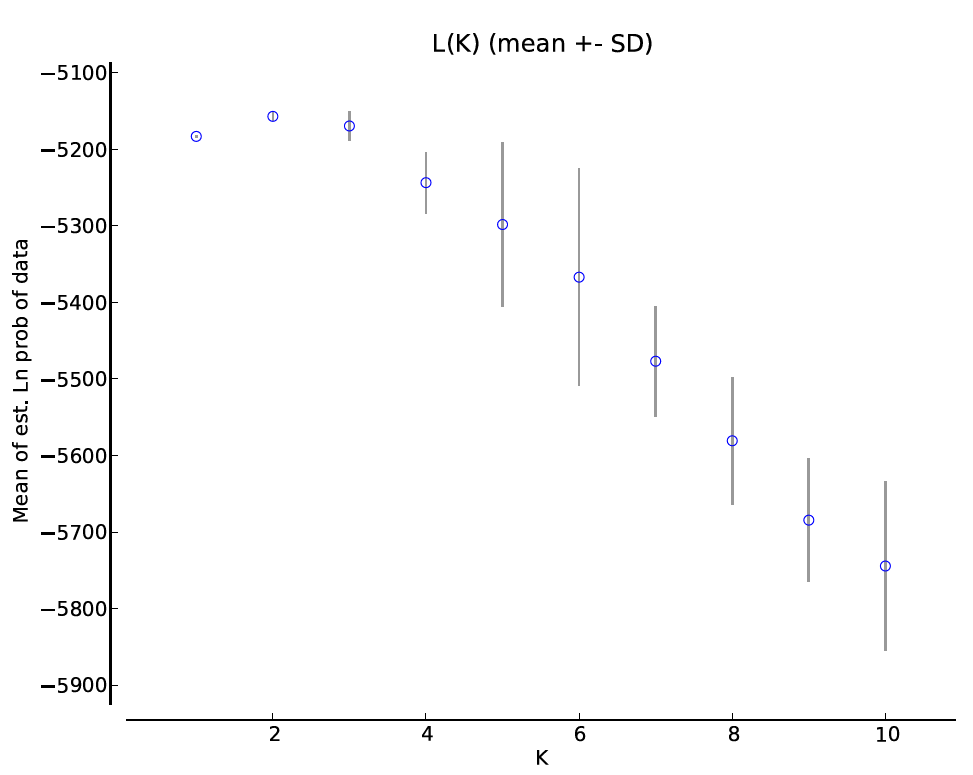

Supplement: Supplementary file 1 — Appendix S1. Genetic diversity of Gentoo penguins (Pygoscelis papua) at fourteen breeding sites across the Scotia Arc. Appendix S2. Maximum clade credibility tree derived from mtDNA showing the origin and differentiation of Pygoscelis papua lineages north (Falkland Islands, light green above) and south of the Polar Front (all other colors and locations). Appendix S3. Graph of the mean likelihood of the number of populations L(K) versus the number of populations K, resulting from STRUCTURE Harvester using data from all study colonies (n = 14), under the Admixture model with Correlated allele frequencies, using No Prior Location in STRUCTURE. Appendix S4. Graph of the mean likelihood of the number of populations L(K) versus the number of populations K, resulting from STRUCTURE Harvester using data from all Falkland Island colonies (n = 10), under the Admixture model with Correlated allele frequencies, using No Prior Location in STRUCTURE. [file ECE3-6-1834-s001.docx]
